# Considerations in Pursuing Reaction Scope Generality

**DOI:** 10.1002/anie.202511091

**Published:** 2025-09-09

**Authors:** Samson B. Zacate, Juliana A. Dantas, Song Lin, Abigail G. Doyle, Matthew S. Sigman

**Affiliations:** ^1^ Department of Chemistry and Chemical Biology Cornell University Ithaca NY 14853 USA; ^2^ Department of Chemistry University of Utah Salt Lake City UT 84112 USA; ^3^ Department of Chemistry and Biochemistry University of California, Los Angeles Los Angeles CA 90095 USA

**Keywords:** Computational chemistry, Generality, High‐throughput screening, Machine learning, Synthetic methods

## Abstract

The term “generality” has recently been popularized in synthetic chemistry, owing largely to the increasing use of high‐throughput technology for producing vast quantities of data and the emergence of data science tools to plan and interpret these experiments. Despite this, the term has not been clearly defined, and there is no standardized approach toward developing a method with a diverse (general) scope. This minireview will examine different emerging strategies toward achieving generality using selected examples and aims to give the reader an overview of modern workflows that have been used to expedite this pursuit.

## Graphical abstract

Synthetic methods that are applicable to a broad range of substrates are sought after, owing to their utility in industrial settings. This minireview describes considerations associated with how chemists define and identify general methods, especially with the emergence of modern analytical, high‐throughput, and data science tools in chemistry, and gives the reader an overview of workflows that have been used to expedite this pursuit.

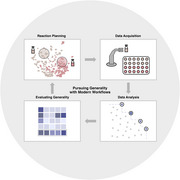

## 1 Introduction

Reaction development is the cornerstone of synthetic chemistry, driven either by practical needs (i.e., promising disconnections for streamlining a target‐oriented synthesis) or by mechanistic design aimed at exploring new reactivity concepts. When disclosing new methods resulting from these goals, chemistry research papers include illustrative examples to demonstrate the reaction scope and inform readers about the method's possible tolerance to substrate deviation. This is in part due to how practitioners optimize reactions, which has historically been focused on a single representative substrate (vide infra). Therefore, the substrate scope serves to help researchers anticipate how a transformation will fare on unseen substrates and in more complex settings—in other words, defining method generality.

Generality conveys how accommodating a synthetic method or catalyst is to diverse substrates while maintaining high levels of yield and selectivity (i.e., enantio, diastero, and site). Although more general methods are clearly considered attractive, how a researcher goes about identifying general reactions is less clear. In our opinion, generality has two main components. The first is how one defines the substrate diversity evaluated for a given method, which may include classic steric/electronic variations, functional group tolerance, and reaction robustness. The second is how one quantifies performance, which could include quantifying the average yield across substates, percentage of reactions that work above a threshold yield value, highest minimum yield across substrates, or perhaps some aggregated metric. Clearly, no method will be perfectly general given the size and diversity of chemical space. Therefore, the goal of this review is not to provide a singular definition of generality in synthetic chemistry but to provide the community with an overview of how chemists both historically and contemporarily discover, define, and assess general reactions and catalysts, as enabled by the emergence of analytical, high‐throughput, and data science tools.

In the context of academic synthetic methods development, there is high value in demonstrating a broad scope to increase the likelihood that methods are applicable in real‐world settings such as medicinal chemistry campaigns or retrosynthetic planning for target‐oriented syntheses. In complex target synthesis, for example, researchers must often predict the performance of reactions with previously unseen substrates, and incorporating general reactions into route planning can improve the chances of success. Once a route has been identified and established, a reaction is then optimized specifically for a single substrate while performance on other compounds becomes less relevant. In this regard, data‐science tools have been used to evaluate both the generality^[^ ^1^ ^]^ and substrate‐dependent responses^[^ ^2^ ^]^ of a proposed reaction to help practitioners prioritize routes with the highest anticipated success rate. These predictive models have been applied in algorithms highlighted in this review as well as reported retrosynthesis platforms.^[^ ^3^, ^4^ ^]^

Given these long‐recognized values, we begin this review with a historical examination of generality, guided by key technological advancements for assessing reaction scope. We then provide an overview of recent strategies for algorithmic selection of scope and machine learning/data science tools that can be integrated into generality‐driven optimization campaigns.

## 2 Enabling Technology and Established Approaches

**Figure 1:**
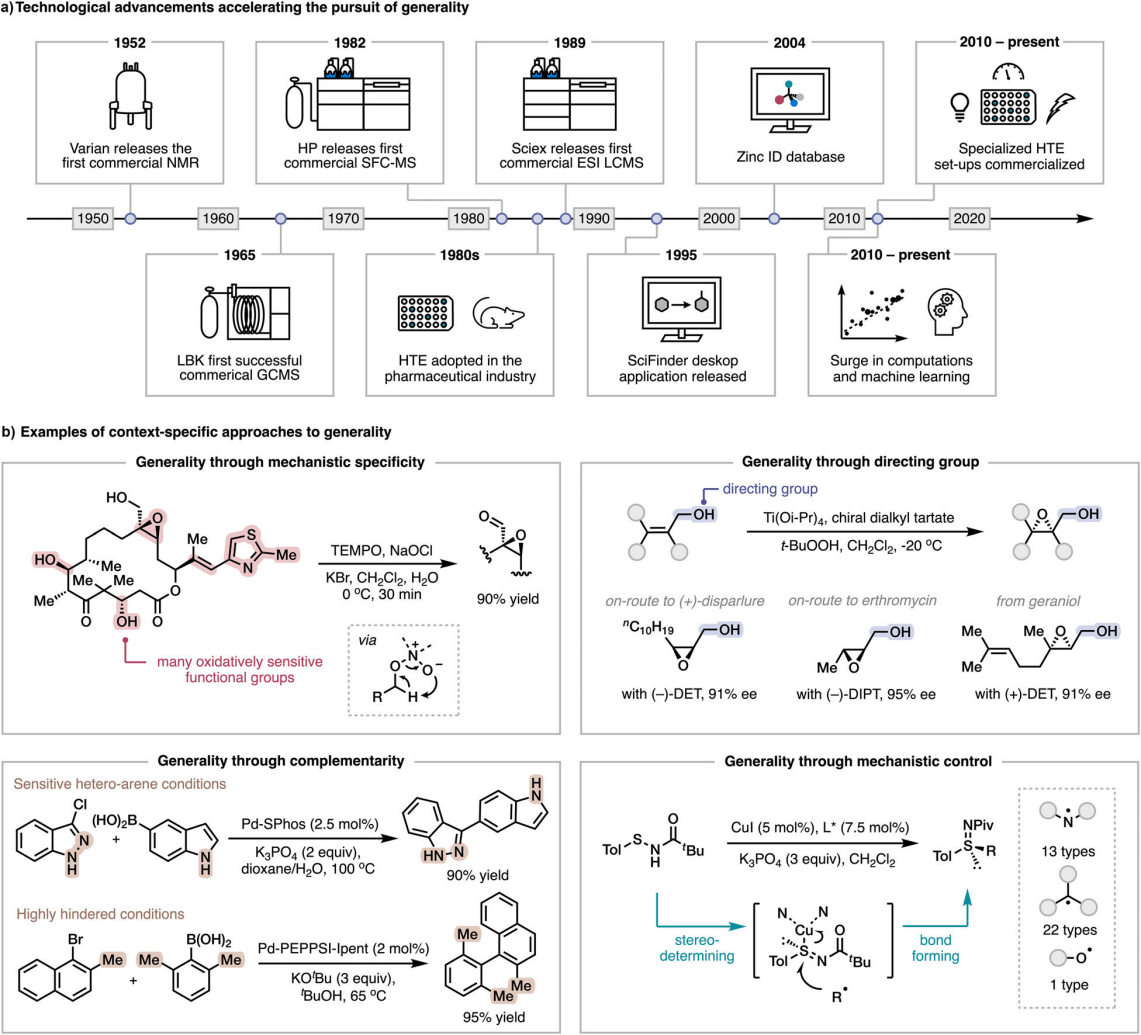
a) Timeline of technological advances that have enabled modern generality‐oriented optimizations. b) Representative examples of context‐specific approaches towards generality.

The prospect of identifying general reaction conditions and catalysts in the 21st century has been greatly impacted by advances in reaction optimization. To appreciate modern optimization workflows, we first reflected on how methods were optimized in the 20th century and the technological advances that have recently emerged to allow for the analysis of large quantities of data for modern generality‐oriented optimizations (Figure 1a). After synthesis of the requisite starting materials, the major bottleneck in a 20th century reaction optimization campaign was the time required to acquire data to assess reaction performance. Interestingly, this remains the rate‐limiting step in the modern day, with advances in technology for reaction set‐up and data analysis outpacing advances in data acquisition (vide infra). Before the commercialization of nuclear magnetic resonance (NMR) spectrometers in 1952, it could take weeks or months for chemists to structurally characterize new products. Over the next few decades, the development of gas chromatography‐mass spectrometry (GC‐MS), supercritical fluid chromatography‐mass spectrometry (SFC‐MS), and liquid chromatography‐mass spectrometry (LC‐MS), which can first separate complex mixtures via chromatography and then identify components via mass spectrometry, were key to decreasing data acquisition time (often minutes). These techniques also required less analyte, enabling the miniaturization of reaction screening. Accordingly, industrial chemists in the 1980s began to adopt well‐plate based high‐throughput experimentation (HTE) to enable the parallel screening of hundreds of compounds for pharmaceutical optimization campaigns. In recent years, specialized reactors such as photochemistry, ^[^ ^5^ ^]^ electrochemistry,^[^ ^6^, ^7^ ^]^ and pressure reactors^[^ ^8^ ^]^ have been developed to enable high‐throughput experimentation under a greater variety of conditions. Furthermore, autonomous robots that can pursue exploratory synthetic chemistry without the direct involvement of an experimental chemist are being developed.^[^ ^9^ ^]^

The rate of achieving reaction generality was also accelerated by the availability of larger and more diverse pools of on‐demand commercial compounds. Historically, due to limited supplies and the time it took to order and receive new chemicals, substrate scopes would often be constructed from the chemicals that were on hand, which limited the understanding of broader compatibility and thus method generality. Moreover, before personal computers became commonplace, researchers relied on shared copies of journals at the library to stay apprised on the literature and would laboriously browse pages of chemical abstracts in search of specific information. In 1995, the release of SciFinder enabled facile access to previously reported reaction conditions, lowering the barrier to building upon existing work. More recently, chemical databases such as Zinc ID, which contain the three‐dimensional structures and properties of billions of commercially available compounds,^[^ ^10^ ^]^ have empowered computational screening efforts.

Of course, modern analytical techniques, robotic equipment, and vast catalogs of commercial chemicals are not mandatory for achieving generality, and technological limitations should not discourage researchers from undertaking a generality‐oriented optimization. The innovations that enable generality do not always result from screening throughput. Instead, context‐specific approaches can be executed to achieve generality by rational design strategies. One such approach is to use functional group handles that rapidly react with one another but are otherwise inert in most contexts (e.g., alkyne and azide in click chemistry^[^ ^11^ ^]^) to allow for high functional group tolerance. In contrast to leveraging specificity by inertness, generality can also be achieved by mechanistic specificity, such as in TEMPO‐catalyzed alcohol oxidations (Figure 1b, top left). In this system, the formal oxidation is achieved via a Cope‐type elimination from a catalyst‐substrate complex rather than alternative electron‐ or hydride‐transfer pathways, thus giving excellent chemoselectivity even in complex settings with several oxidatively labile functionalities. ^[^ ^12^, ^13^ ^]^ Another common strategy is to use a directing group that interacts with a reagent or catalyst to control the location or orientation of a reaction.^[^ ^14^ ^]^ This strategy implies that if an identical directing group is incorporated into a new substrate, then the transformation should proceed in the same manner, regardless of the composition of the rest of the molecule. For example, in the Sharpless asymmetric epoxidation^[^ ^15^, ^16^, ^17^ ^]^ (Figure 1b, top right), an allylic alcohol coordinates to the active Ti‐tartrate complex to direct the attack by a catalyst‐bound peroxide to a specific face of the alkene, resulting in a highly enantioselective reaction across a range of substrates with varying degrees of alkene substitution.

In many cases, it may not yet be feasible to establish a single truly general method. In these instances, a set of complementary conditions may allow access to a broader substrate scope. Notably, when establishing this set, simply combining all top performing conditions often does not result in optimal generality as lower‐coverage conditions frequently complement more general conditions.^[^ ^18^ ^]^ A classic example of this is C(sp^2^)‐C(sp)^2^ cross‐couplings, for which the choice of metal, ligand, and reactive functional groups can be varied to best arrive at the desired product.^[^ ^19^ ^]^ For instance, Suzuki–Miyaura coupling conditions that are tailored for highly hindered substrates or sensitive heteroarenes are complementary to more standard conditions and together cover a broader substrate space^[^ ^20^, ^21^ ^]^ (Figure 1b, bottom left). Similarly, many different conditions exist for the activation of carboxylic acid component of amide couplings and can be tailored to the requirements of a specific substrate combination.^[^ ^22^ ^]^ Not surprisingly, both amide couplings and cross‐coupling reactions are at the heart of modern pharmaceutic discovery and development as their generality is enabling. Additionally, the development of new reagents and technology for reaction techniques such as bio‐, electro‐, and photo‐chemistry can provide opportunities for alternative mechanistic pathways to arrive at the same products, which can fundamentally expand the scope of a transformation. For example, traditional Cu‐radical coupling reactions proceed via a stereo‐determining and bond‐forming reductive elimination whose scope generality may be limited by a trade‐off between reactivity and selectivity.^[^ ^23^ ^]^ Gu, Hong, Dong, and Liu et al. overcame this shortcoming by leveraging a copper catalytic system (Figure 1b, bottom right) wherein the initial formation of a Cu(II)‐sulfinimidoyl complex is rendered stereo‐determining and is decoupled from subsequent bond formation, which proceeds via a stereospecific S_H_2 substitution with a breadth of C‐, N‐, and O‐centered radicals.^[^ ^24^ ^]^

## 3 Modern Strategies for Accelerating Generality‐Oriented Optimization

Historically, chemists have optimized reactions by iteratively varying one parameter at a time. After gaining rational insights from these modifications and probing mechanistic hypotheses, conditions can then be improved for a subset of substrates and the process can be repeated until satisfactory performance is achieved across a range of substrates. A limitation of this approach arises when mechanisms for complex systems are not conserved across substrates (i.e., change in rate‐controlling step), which can convolute the use of mechanistic approaches to aid optimization.^[^ ^25^ ^]^ Additionally, in accordance with the adage “you get what you screen for,” most traditional optimizations, which have relied on only a singular model substrate, often are not transferrable to structurally diverse compounds. Moreover, this one‐factor‐at‐a‐time optimization can funnel conditions into a local maximum rather than a global maximum (e.g., base A may be best for an optimization campaign using solvent B, but solvent C, which is usually inferior, could perform much better specifically with base D).

In the 21st century, the predominant approach toward rapidly achieving generality has relied on the acquisition and analysis of larger datasets. Use of HTE with standardized well‐plates enables chemists to run many reactions in parallel (commonly 96), allowing for simultaneous exploration of many reaction variables.^[^ ^26^, ^27^, ^28^ ^]^ HTE facilitates optimization toward global maxima and the generation of a more complete data set, which can help establish the limits of a method and lower the barrier to acquiring negative results^[^ ^29^ ^]^ that are critical for a better understanding of a method and for training machine learning (ML) models. Optimization workflows are further streamlined by compatible HTE systems such as preplated reagent libraries, multichannel pipettors, liquid and solid handling robots, and stirring systems, with new analytical approaches actively being developed to match the rate of data generation. Despite these advances, high‐dimensional combinatorial optimization campaigns with multiple substrates and reaction components remain impractical or not possible in many cases. To overcome this challenge, innovative practical strategies for generality‐oriented, multiple‐substrate optimization campaigns have been developed using a broad range of computational, statistical, and ML tools.^[^ ^30^, ^31^, ^32^, ^33^, ^34^, ^35^ ^]^ With these modern techniques available,^[^ ^36^ ^]^ there are numerous practical choices a user must make, including how to apply ML for varied objectives in synthetic chemistry optimization. The following section is structured based on the order of tasks in reaction optimization: first substrate selection, then data acquisition, and finally data analysis (whereupon the process may be repeated) and includes discussion on the strategies and technologies that have been applied in the pursuit of generality at each stage. While there is not yet consensus on the “best” approach, the resulting synthetic methodologies are without a doubt more general due to the application of these tools.

### 3.1 Substrate Selection

To identify general conditions, one must test conditions against a wide variety of substrates, but how do you define and identify this “wide variety”? Qualitatively, variety in the context of functional group diversity would consist of surveying substrates with electron‐rich/poor, acid/base‐sensitive, and redox‐active moieties. More broadly, steric and electronic variability as well as applicability to specific use cases (e.g., pharmaceutically relevant motifs) should be tested. However, human chemical intuition alone is likely insufficient at capturing an entire map of substrate space and is biased to select substrates that are more likely to perform well. To overcome this challenge, researchers have been applying data‐driven modeling for synthetic chemistry since the last century, exploring techniques that originated from linear free energy relationships (LFERs) to correlate molecular properties with reaction performance using multivariate linear regression.^[^ ^37^, ^38^, ^39^ ^]^ Chemical and fragment databases^[^ ^40^, ^41^ ^]^ can be computationally generated by encoding structural information into numerical or categorical variables called molecular descriptors (e.g., buried volume, polarizability, natural bond orbital). These structural descriptor libraries can then be used to generate two‐dimensional representations of chemical space (chemical space maps) in order to visualize and, in the context of this review, select “diverse” substrates to survey generality.^[^ ^42^ ^]^ This tool has also been broadly utilized for condition screening and for building predictive statistical models in reaction optimization.^[^ ^43^ ^]^

The visualization of chemical space maps is achieved via dimensionality reduction techniques [e.g., principal component analysis (PCA),^[^ ^44^ ^]^ uniform manifold approximation and projection (UMAP)^[^ ^45^ ^]^ and t‐distributed stochastic neighbor embedding (t‐SNE)]^[^ ^46^ ^]^ and clustering by an appropriate algorithm (e.g., HDBSCAN, DBSCAN, K‐Means, GMM, Ward, and Birch)^[^ ^47^ ^]^ that groups similar compounds by structural features, properties, or reaction output. The choice of technique is nuanced, but PCA is utilized when a defined linear behavior among the variables (features) is demonstrated by transforming the new coordinate systems into new axes (principal components) containing the greatest variance in the data by orthogonal vectors representation. When using interpretable parameters, PCA is considered adequate for chemical space map generation if meaningful datapoint distribution and similarity (as defined by the user) of the grouped structures is observed. If not, a nonlinear method is explored; UMAP and t‐SNE are differentiated by global or local preservation of similarity with UMAP providing outstanding performance in preserving global structure. While user‐defined, evaluation of dimensionality reduction typically focuses on class separation, risk of overfitting, and dataset size. However, the choice of technique is also reliant on how many features and compounds are analyzed, and we point the reader to the primary literature for further advice.^[^ ^48^ ^]^

These maps have been applied to guide scope selection from a large pool of commercially available or bioactive compounds collected from chemical databases.^[^ ^49^, ^50^, ^51^ ^]^ The list of structures can be filtered by commercial availability or synthetic viability, functional groups, molecular mass range, number of fragments (neutral molecule or salt), lipophilicity, or other properties, allowing for a precise representation of chemical space. Beyond visualizing chemical space, molecular descriptors that can capture the influence of substituents, the entire molecule, or a specific reactive functional group often aid mechanistic understanding and reaction optimization.^[^ ^52^, ^53^ ^]^ Correlation of these descriptors with reaction outcomes [e.g., yield, selectivity, or rates] enables the quantification of structure‐activity relationships to provide a deeper understanding of the system and predict reactivity.^[^ ^54^ ^]^ However, constructing descriptor libraries to capture conformational sampling using computationally expensive methods can limit access and applicability of the approach.^[^ ^55^ ^]^ To address these issues, the creation of open‐access descriptor databases ^[^ ^56^, ^57^, ^58^ ^]^ and more recently the prediction of conformationally‐dependent descriptors^[^ ^59^ ^]^ have been introduced.

**Figure 2:**
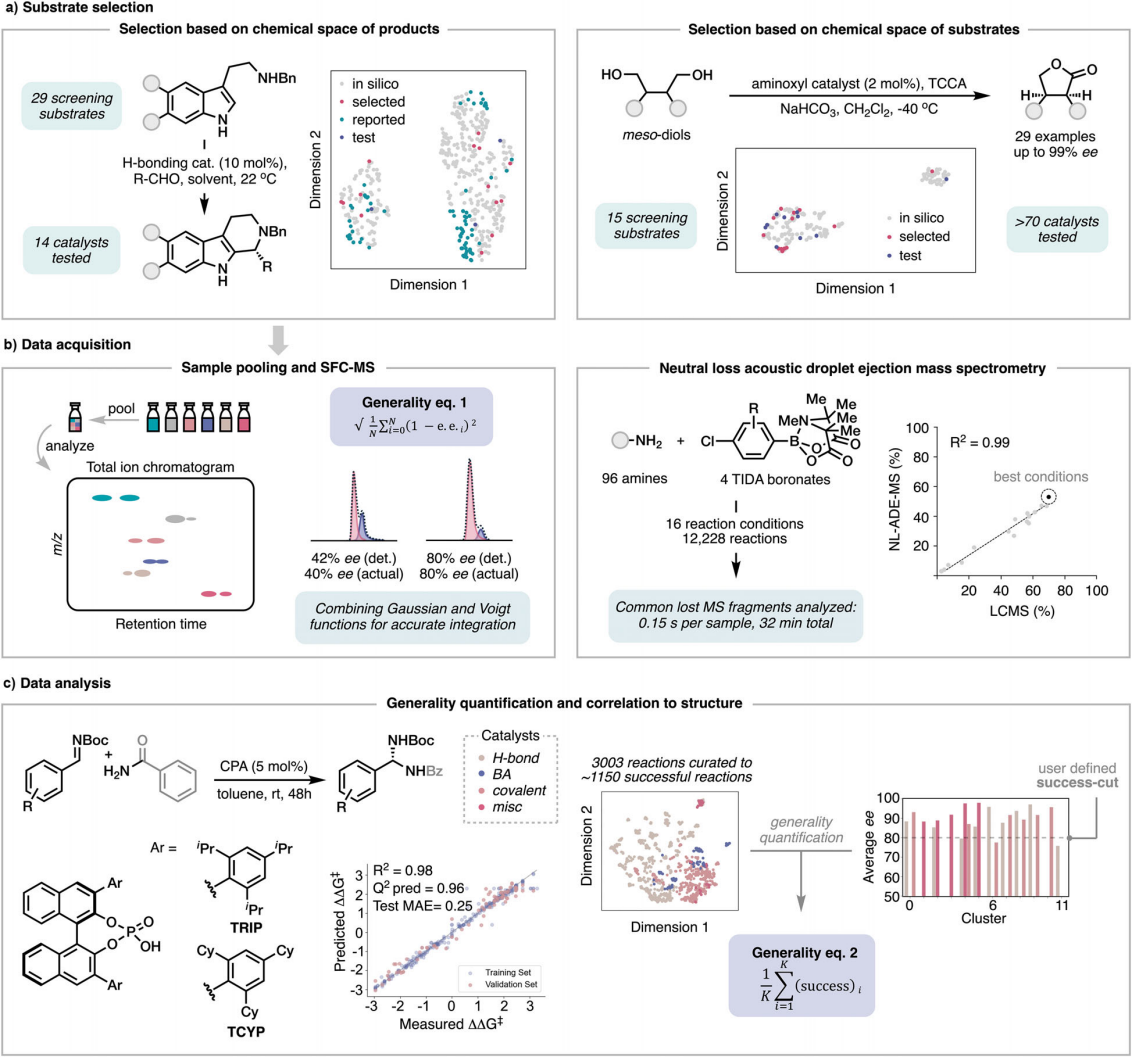
a) Examples of substrate selection guided by computational tools. b) Examples of data acquisition strategies for probing a large number of reactions. c) Example of a workflow to assess and quantify generality toward identifying a general catalyst.

The utility of computational tools can be augmented by employing a hybrid approach that combines data‐driven substrate selection with decision‐making enhanced by human researchers. As an example, Jacobsen and coworkers identified a general catalyst for enantioselective Pictet–Spengler reactions by leveraging data‐driven substrate selection techniques^[^ ^60^ ^]^ (Figure 2a, left). To identify diverse screening combinations, they constructed a virtual library of 340 potential products and performed UMAP dimensionality reduction to generate a chemical space representation. To select substrates, the authors used a custom implementation of k‐means clustering in which certain cluster centers of interest could be predefined. The recommendations of product clustering were balanced with practical considerations, such as the commercial availability of the substrates, allowing for selection of compounds encompassing regions of chemical space that were largely unexplored by the previously reported methodologies.

Sigman, Miller, and Lin et al. generated a representation of the chemical space of all commercially available 1,4‐*meso*‐diols for substrate selection towards developing a general catalyst for the preparation of chiral lactones (Figure 2a, right).^[^ ^61^ ^]^ Notably, the initial set of diols revealed that the commercial substrates were overpopulated by compounds containing similar pyrrolidine substructures. To enable analysis of other substructures, a representative pyrrolidine substrate was selected, and a truncated dataset was used, which partitioned the substrates into di‐substituted (left cluster) or tetra‐substituted (right cluster) diols. From this, 14 structurally diverse substrates with a variety of functionalities were selected with an additional 1,5‐diol included to further increase diversity in the optimization.

An alternative approach to achieve generality is by determining functional group compatibility. Glorius et al. formalized a “robustness” screen to rapidly test reaction scope by assessing the compatibility of a reaction to additives bearing a broad variety of functionalities.^[^ ^62^, ^63^ ^]^ The advantage of this approach is that acquisition of new substrates and analysis of new products is not required; however, it is possible that false positive or negative results can arise from differences in reactivity when incorporating functional groups into the substrates themselves. In an approach that predated the data‐driven design of reaction scopes using the unsupervised learning techniques describe above, researchers at Merck used PCA to identify and standardize relevant sets of complex drug‐like molecules that they termed “informer libraries” for testing the compatibility in cross‐coupling reactions ^[^ ^64^ ^]^ and this approach was later extended to other classes of reactions by Glorius and coworkers^[^ ^65^ ^]^ In addition to visualizing substrates, understanding the chemical space of other parameters is also useful for screening aimed at identifying general reaction conditions. For example, a plot of solvent dipole moment vs. dielectric constant can be helpful to test multiple solvents within a specific class (e.g., polar protic) or to cover many different solvent classes.^[^ ^26^ ^]^

### 3.2 Data Acquisition

Given the accessibility of high‐throughput reaction set‐ups and planning as outlined above, innovation in methods for data acquisition is critical for overcoming the throughput limitation of time‐intensive chromatography for reaction analysis. In 1998, Kagan and coworkers disclosed the use of multiple pooled substrates for screening against a single catalyst in one pot to accelerate throughput for the optimization of an asymmetric ketone reduction.^[^ ^66^ ^]^ In fact, they boldly deemed it “embarrassing” to only select a representative single‐model substrate to test a new catalyst. Though innovative, their approach was limited by the resolution achievable by HPLC separation for analysis of multiple products. More recently, List and coworkers used a similar pooled substrate screening approach for the identification of a general Diels–Alder catalyst but again the substrate selection and quantity were limited by the capability of GC separation.^[^ ^67^ ^]^

Jacobsen et al. addressed these limitations by combining sample pooling and SFC‐MS analysis (Figure 2b, left),^[^ ^60^ ^]^ using extracted ion chromatograms to determine the *ee* of multiple products based on their differing molecular weights even when they co‐elute. Moreover, the magnitudes of MS signals are solvent‐independent, which allows a single, generic gradient to be applied for analytes over a wide range of polarities. Given that baseline separation of all enantiomeric pairs would not always be achieved with a single run, they also developed a peak‐fitting method to extract accurate integrations from partially separated enantiomers. To assess the generality for each catalyst, the authors used a generality metric which summarizes the collection of enantioselectivity values into a number between 0 and 1, where 1 would represent a completely general catalyst that induces 100% *ee* in every reaction.

(eq. 1) $$\begin{array}{c} \text{generality informed by average outcome}=\\ \surd \frac{1}{N}\sum_{i=0}^{N}{\left(1-e.e._{i}\right)}^{2} \end{array}$$

Hartwig et al. disclosed a strategy for deconvoluting complex MS spectra, which allowed for analysis of pooling‐based HTE by assessing three pools of compounds that shared the same reactive functional group but carried inactive substituents with different masses. This approach enabled simultaneous identification of products based on their unique differences in masses.^[^ ^68^, ^69^ ^]^ In addition to substrate pooling strategies, there has been much recent work on shortening the analysis time of single samples. Ouyang et al. reported determination of enantioselectivity with MS by inducing directional rotation of chiral gas‐phase ions, obviating the need for chromatographic separation and shortening reaction analysis to less than one minute.^[^ ^70^ ^]^ Substrate design can also aid in decreasing analysis time, although it is less widely applicable. Chen and coworkers were able to rapidly assess *ee* by performing a post‐reaction click‐chemistry product derivatization to form diastereomers, which could then be differentiated by ion mobility spectroscopy. ^[^ ^71^ ^]^ HBlair and coworkers performed a generality‐oriented optimization of the Pd‐catalyzed cross‐coupling of an amine with halo‐TIDA boronates using neutral loss acoustic droplet ejection mass spectrometry (NL‐ADE‐MS) (Figure 2b, right).^[^ ^72^ ^]^ Since all products contained the same boronate unit that undergoes fragmentation to release methyl acrylic acid during mass spectrometry analysis, quantification of this shared fragment could be used to determine yield regardless of the molecular weight and ionizability of the remainder of the molecules. The determined yields were in excellent agreement with LC‐MS analysis and allowed for the analysis of 12,228 reactions in only 32 min.

In contrast to the data‐driven selection method described in the previous section, rapid sample analysis can also allow for serendipitous optimization. For example, Dreher and MacMillan et al. screened 721 additives using a liquid handling robot and UPLC‐MS analysis to improve the scope of a decarboxylative arylation reaction.^[^ ^32^ ^]^ They identified that the addition of phthalimide uniquely improved reactivity for unactivated carboxylic acids by acting as a stabilizing ligand to both prevent the decomposition of Ni‐aryl complexes and reactivate off‐cycle multimeric Ni species. These interactions would have been challenging to predict by rational design.

### 3.3 Data Analysis

Qualitatively, a reaction is accepted to be general if high performance is achieved with a diverse ensemble of substrates, but how do we quantify generality? Reid and coworkers reported a catalyst‐agnostic workflow centered on unsupervised machine learning to assess and quantify catalyst generality for enantioselectivity based on reaction conditions already reported in the literature (Figure 2c).^[^ ^73^ ^]^ The workflow started with the curation of 3003 reported organocatalyzed Mannich reactions with varied catalytic modes of activation (e.g., H‐bonding, Brönsted acid, covalent, and miscellaneous catalysts). Nonlinear dimensionality reduction and unsupervised clustering were performed, leading to an extensive chemical space for a set of reactions containing a similar distribution of properties. With this distribution map compiled, generality values can be quantified by measuring the extent of substrate chemical space covered by the catalysts in each cluster based on enantioselectivity values that are deemed as acceptable, with *K* being the total number of clusters in the chemical space and *success* being the average performance higher than the threshold defined. From this analysis, information per cluster regarding catalyst performance, frequency of use, and structural diversity can be extracted.

(eq. 2) $$\begin{array}{c} \mathrm{generality}\;\mathrm{info}\mathrm{rmed}\;\mathrm{by}\;\mathrm{outcome}\;\mathrm{and}\;\mathrm{chemical}\;\mathrm{structure}\;=\\ \frac{1}{K}\sum_{i=1}^{K}\left(\mathrm{success}\right)i \end{array}$$

After establishing a generality score, the authors focused on examining a specific catalyst chemotype (chiral phosphoric acids) to correlate substrate and catalyst structure with generality performance. Values of ΔΔ*G* ^‡^ for multiple types of reactions were extracted as output to identify a correlation from calculated substrate and catalyst features in a multivariate linear regression (MLR). From this analysis, TRIP and TCYP were predicted to be the most general chiral phosphoric acid catalysts based on values of a steric descriptor, B5 Sterimol. Both TRIP and TCYP were then tested on a variety of reactions and both displayed high generality and complementarity, despite the fact the TCYP is 8‐fold less utilized in the literature.

### 3.4 Optimization Platforms

ML can enable resource‐efficient optimization with many reaction parameters beyond that achievable by intuition‐driven high‐throughput experimentation, especially when considering multiple objectives, such as yield and selectivity (regio‐, site‐, enantio‐, chemo‐) simultaneously. However, the application of algorithmic optimization tools is not directly suited to the identification of general conditions since an optimizer will seek to identify optimal substrates as well as conditions in order to maximize the objectives (high yield/*ee*). Instead, since algorithmic tools adapted for generality consider aggregate performance across multiple substrates, they must predict not only the performance of untested conditions for each substrate but also how a condition tested on one substrate will perform on the remaining, untested substrates. The work discussed in this section will introduce the reader to recent advances in ML‐guided reaction optimization and applications of these approaches to the identification of general reaction conditions across a scope of substrates.

**Figure 3:**
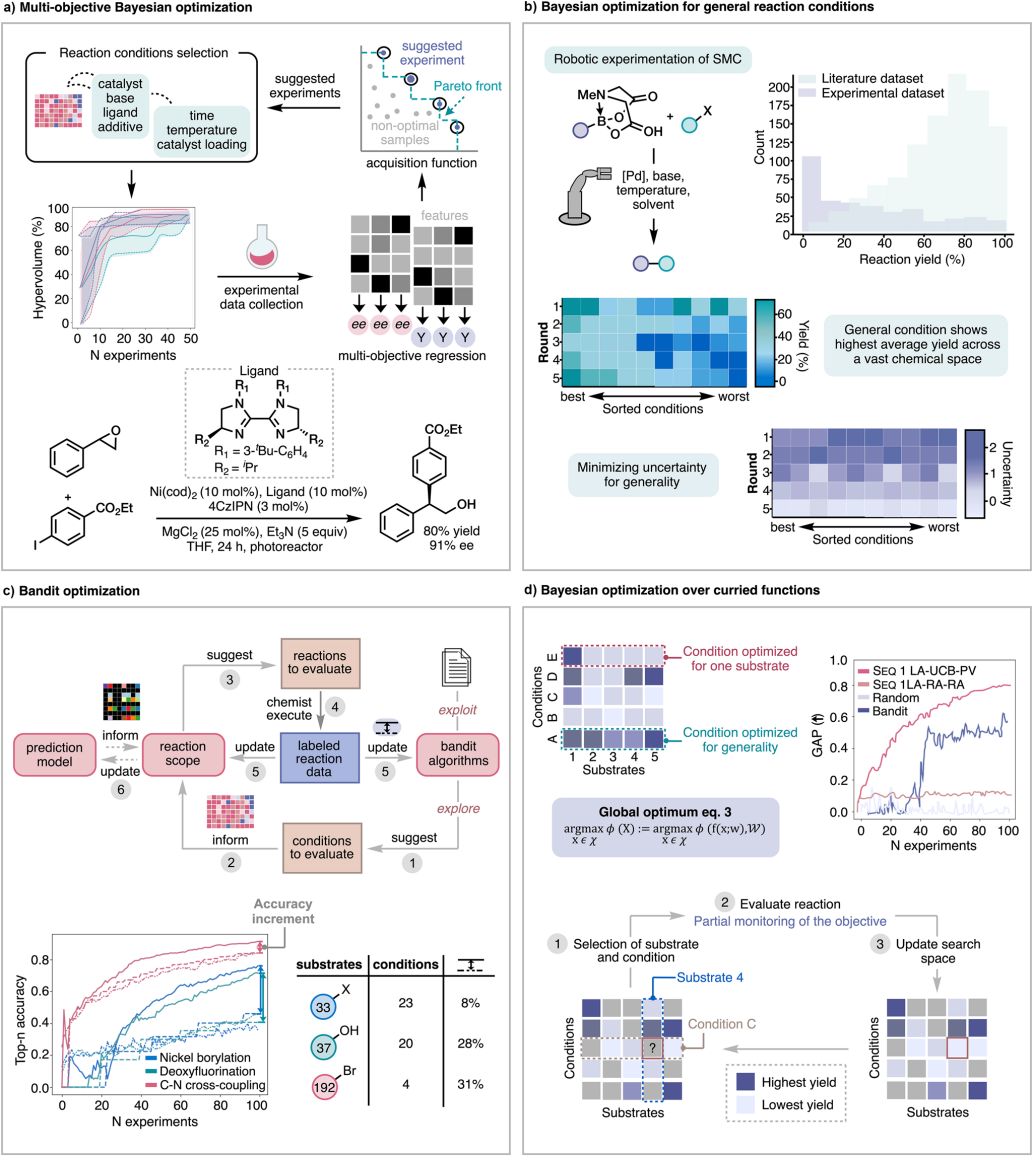
a) Multiobjective reaction optimization via BO. b) Application of BO toward identifying general reaction conditions. c) Identification of general reaction conditions using Bandit optimization models. d) Generality‐oriented optimization as an optimization problem over curried functions.

Bayesian optimization (BO)^[^ ^74^, ^75^, ^76^, ^77^ ^]^ is a strategy that has been applied to efficiently identify global maxima in reaction condition optimization campaigns in lieu of one variable at a time optimization or design of experiments (DOE). Although it was initially disclosed as an algorithm applied for accelerating reaction condition searches for an individual/specific chemical reaction, it has recently been applied as a tool for general conditions search. Before we delve into modification of BO to query generality, we first provide nonexpert readers with foundational details about multiobjective reaction optimization via BO. (Figure 3a). Doyle and coworkers reported the development of an open‐source platform, experimental design by Bayesian optimization (EDBO+), that allows chemists to integrate state‐of‐the‐art optimization algorithms into their everyday laboratory practices.^[^ ^78^ ^]^ BO is an uncertainty‐guided response surface method that minimizes the number of experimental observations necessary to find a global maximum in a search space. To start the workflow, the chemist needs to identify an initial set of parameters [e.g., catalysts, temperatures, and concentrations] to be explored in the optimization of a given reaction, delineate the objectives to be optimized (e.g., yield, *ee*, *rr*), and decide the number of experiments to be evaluated in parallel (batch size). It can also be helpful to obtain calculated descriptors for each discrete component of the reaction to improve optimization efficiency and interpretability. Initialization of the optimization campaign can occur using already existing data or by algorithmic selection, such as via a random seed, Latin Hypercube sampling, or Centroidal Voronoi Tesselation, which seek maximally different initial conditions. The chemist then performs the suggested experiments and enters the results as outputs back into the dataset. BO uses the experimental data to build a regression model, known as a surrogate function, that predicts the objectives (e.g., yield, *ee*, TOF etc) for the virtual space of remaining untested conditions and BO suggests untested conditions for experimental evaluation based on another algorithm, known as an acquisition function, which seeks to balance exploitation of existing experimental outcomes with the uncertainty of the surrogate model predictions.^[^ ^79^ ^]^ The authors tested this workflow for the enantioselective synthesis of 2,2‐diarylalcohols. From a space of thousands of possible scenarios, BO suggested experiments that ultimately identified conditions with improved yield (80%) and enantioselectivity (91% *ee*) in the first 24 reactions, surpassing the originally identified conditions which afforded 70% yield and 91% *ee* obtained after running 500 experiments via one‐factor‐at‐a‐time screening.

Grzybowski and Burke et al.^[^ ^80^ ^]^ reported a closed‐loop workflow with Bayesian optimization that sought general conditions for (hetero)aryl Suzuki–Miyaura cross‐couplings (SMC) and was the first example of data‐driven experiment planning toward finding general conditions (Figure 3b). To reduce experimental effort, a down‐selection of commercially available building blocks for this transformation was driven by dimensionality reduction via the t‐SNE technique and clustering using a stratified clusterization strategy algorithm. The compounds were clustered by their common (hetero)aromatic ring substructures, utilizing Tanimoto's similarity as one of the criteria. The most representative example was selected from the centroid of each cluster.^[^ ^81^ ^]^ Four condition dimensions were considered (3 solvents x 2 bases x 2 temperatures x 4 catalysts) with 11 pairs of substrate combinations of halides and MIDA‐boronates, which maximized mutual dissimilarity of the resulting products. The closed‐loop rounds allowed them to observe how the model improves by decreasing uncertainty as the algorithm learns reaction‐condition space via probing both low and high yielding conditions (whereas low yielding conditions were underrepresented in the literature). Here, the scenario differs from standard BO in that there is more than one substrate that needs to be evaluated but every substrate is not experimentally tested against every condition. Instead, in order to account for generality, the performance for a given condition was estimated not only based on experimental data from tested substrates but also by predicted outcome of the remaining untested substrates under that condition. The authors further improved their workflow in this study by using an automatic robotic system that ensured highly reproducible reactions yields throughout this work. Their best conditions were then tested against 20 dissimilar substrate pairs unseen by the model, providing 95% successful transformations and doubling the average yield compared to benchmark conditions.

Wang et al. recently sought to develop an approach to identify general reaction conditions in a data‐efficient manner by implementing and applying reinforcement learning Bandit optimization models (Figure 3c).^[^ ^82^ ^]^ In this workflow, the chemist defines the conditions and substrates that they want to consider, using, for example, the reaction planning tools outlined in section 3.1. The goal of the algorithm is to identify which condition is most general (as defined by the user) for that scope of substrates without testing all conditions on all substrates. Like other active learning approaches, Bandit algorithms seek to balance exploration of untested conditions and substrates with exploitation of tested successful conditions, while also considering that different substrates lead to different outcomes. Importantly, the substrate search space for this model does not need to be static, meaning that the scope can be changed on the fly as part of the feedback from the environment. The Bandit optimization models were simulated using three previously published reaction datasets: a nickel‐catalyzed borylation, an alcohol deoxyfluorination, and a Pd‐catalyzed C–N cross‐coupling reaction. The simulations were used to determine how many data were required to reach high accuracy in identifying the most general conditions for those three reactions and to compare the efficiency of Bandit optimization to that of baseline algorithms which mimic state‐of‐the‐art multisubstrate screening approaches. The Bandit algorithm showed significant improvements in accuracy of correctly identifying the most general conditions compared with control baselines at 100 experiment budgets for all three case studies (8%, 28% and 31%). In general, it was found that the upper confidence bound (UCB) algorithm reached >80% accuracy of identifying the most general conditions after surveying 10% of the search space; thus, this algorithm and experimental budget was recommended for new optimization campaigns. The efficacy of the Bandit algorithm was further substantiated through external validation with unseen chemical transformations. No pre‐training or data was needed for these new applications, which shows that Bandit optimization applies to diverse chemistries and can optimize numerous conditions (continuous and categorical).

Schmid, Aspuru–Guzik, Kristiadi, Jorner, and Strieth–Kalthoff et al. recently systematically evaluated different algorithmic approaches for generality‐oriented optimization, which they formalized as an optimization problem over curried functions for a more direct comparison of the methods (Figure 3d).^[^ ^83^ ^]^ They define the objective as maximizing a user‐defined generality metric called the aggregation function *ϕ*, for which *x* are conditions and *w* are substrates.

(eq. 3) $$\begin{array}{c} \mathrm{global}\;\mathrm{optimum}\;=\\ \underset{X\in \chi }{\arg\max\;\phi \;(X)}:=\underset{X\in \chi }{\arg\max\;\phi (f(x;w),W}) \end{array}$$

The optimization is modeled as a curried function approach in that the function (generality objective) takes multiple arguments (reaction parameters), but instead of taking them all at once, it takes them one by one. In other words, the outcome of the recommended experiment corresponds to a partial observation of the generality objective, which needs to be taken into account when recommending the next experiment. The authors used four HTE reaction optimization data‐sets as benchmarks to evaluate previously reported algorithms, augmenting the search space to incorporate larger amounts of negative results due to the bias of HTE datasets toward higher yield data. They found that the BO‐based strategy (SEQ 1 LA‐UCB‐PV) outperformed the Bandit algorithm, although both methods readily outperformed random base‐line algorithms. Further, modification of the algorithms revealed that while more explorative selection of conditions improves performance, the method of substrate selection does not significantly influence optimization and that one‐step lookahead acquisition functions are well‐suited for generality‐oriented optimization of chemical reactions, performing on par with more complex approaches.

## 4 Outlook

It is evident that there is an emerging driving force from chemists to develop synthetic methods that are more general, and thereby more applicable to new use cases. Efforts have previously focused on expanding the number of hand‐selected substrates in scope tables, additive screening campaigns, and late‐stage functionalization examples. All of these have helped chemists identify and assess the generality of new methods but have come with limitations. The use of data science tools and automation is a relatively recent development to address these goals, offering benefits such as reducing human bias and the number of experiments required to identify a satisfactory solution. However, there are clearly many more opportunities to improve these approaches and develop alternatives to make the search for generally applicable reactions more accessible to practicing chemists, suitable for distinct types of chemistries, and data efficient. These tools are contemporary and have not seen application in many real‐world optimization campaigns yet, which makes it challenging to fully understand their strengths and limitations. Real‐world use cases will be crucial to further evaluate, compare, and enhance these new strategies. Nevertheless, it is clear that, combined with data‐driven substrate selection, they offer a deliberate approach to identifying general conditions during optimization, which model system‐based optimization does not, and they can reduce the number of experiments necessary to identify conditions compared with full combinatorial screening via HTE.

Although there is no standardized or correct strategy to optimize for generality, focusing on structures with a specific functional objective (e.g., bioactivity, energy density) is often a good start for defining a method useful in real‐world industrial applications. Along these lines, we suggest avoiding the use of only high performing “privileged substrates” that may be convenient for achieving good results but are not representative for broader applications. Furthermore, to effectively train optimization algorithms and inform practitioners of the limitations of reaction methods, it is crucial to publish results that are traditionally considered as negative or poor. We look forward to seeing new strategies emerge and challenge how chemists pursue generality‐oriented optimization.

## Conflict of Interests

The authors declare no conflict of interest.

## Data Availability

Data sharing is not applicable to this article as no new datawere created or analyzed in this study.
